# Investigating Vitamin D-Binding Protein’s Role in Childhood Health and Development

**DOI:** 10.3390/ijms25116272

**Published:** 2024-06-06

**Authors:** Charlotte Delrue, Reinhart Speeckaert, Joris R. Delanghe, Agnieszka Prytuła, Marijn M. Speeckaert

**Affiliations:** 1 Department of Nephrology, Ghent University Hospital, 9000 Ghent, Belgium; charlotte.delrue@ugent.be; 2 Department of Dermatology, Ghent University Hospital, 9000 Ghent, Belgium; reinhart.speeckaert@uzgent.be; 3 Department of Diagnostic Sciences, Ghent University, 9000 Ghent, Belgium; joris.delanghe@ugent.be; 4 Department of Pediatrics, Ghent University Hospital, 9000 Ghent, Belgium; agnieszka.prytula@uzgent.be; 5 Research Foundation-Flanders (FWO), 1000 Brussels, Belgium

**Keywords:** children, polymorphisms, vitamin D-binding protein

## Abstract

Vitamin D-binding protein (DBP), also known as Gc-globulin, is a protein that affects several physiological processes, including the transport and regulation of vitamin D metabolites. Genetic polymorphisms in the *DBP* gene have a significant impact on vitamin D levels and may have implications for disease risk. *DBP* polymorphisms are linked to differential immune responses, which could influence the onset of juvenile diseases. This narrative review examines the various roles of DBP, with a focus on bone health, immunological regulation, and lipid metabolism in children. Chronic disorders affected by *DBP* polymorphisms include bone abnormalities, autoimmune diseases, cardiovascular issues, childhood asthma, allergies, cystic fibrosis, acute liver failure, celiac disease, inflammatory bowel disease, and chronic kidney disease. Future research should focus on identifying the processes that underpin the many roles that DBP plays and developing customized therapeutics to improve health outcomes in the juvenile population.

## 1. Introduction

Vitamin D is essential for the development of children, particularly bone health. It assists in the absorption of calcium and phosphorus, which are two minerals necessary for bone formation and growth. Vitamin D deficiency can cause rickets, a disorder characterized by bone weakness and skeletal deformities. Aside from its role in bone health, vitamin D is essential for immune function and may help reduce the prevalence of autoimmune diseases [1]. Vitamin D-binding protein (DBP), also called Gc-globulin, is the major transporter of vitamin D metabolites in the bloodstream. It modulates the bioavailability of vitamin D in many tissues. Genetic variations in the *DBP* gene can produce significant inequalities in vitamin D status across children, suggesting that vitamin D delivery should be customized to individual genetic backgrounds to improve health outcomes [2]. *DBP* polymorphisms have been connected to various immunological responses, which may influence the development of juvenile disorders [3]. As the increasing role of DBP in pediatric health is becoming recognized, the present review aims to summarize the multiple roles of DBP in childhood health.

## 2. The Various Functions of DBP

The principal role of DBP is to bind and transport vitamin D metabolites, such as 25-hydroxyvitamin D [25(OH)D] and 1,25-dihydroxyvitamin D [1,25(OH)_2_D], into the bloodstream. DBP also transports the parent molecule, vitamin D itself. Although vitamin D absorbed from dietary supplements is transported in the blood in association with chylomicron lipids or with lipoproteins, the vitamin D formed in skin cells by the action of ultraviolet radiation on 7-dehydrocholesterol is only released from those cells and transported into the blood by binding to extracellular DBP. As skin synthesis of vitamin D is the main source of vitamin D, the role of DBP in collecting vitamin D for its subsequent metabolism in the liver is a critical aspect of DBP function [4]. Moreover, this transportation is essential because it controls the bioavailability of vitamin D, which is required for calcium absorption, impacting bone health and mineral balance (Table 1).

**Figure 1:**
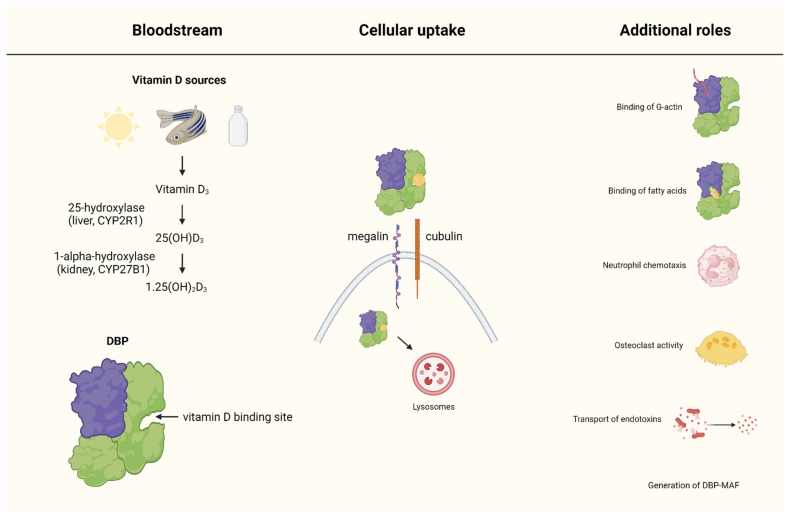
Role of vitamin D-binding protein (DBP) in vitamin D metabolism and bioavailability, and additional roles.

DBP is incorporated into various cell types through interactions with the cell membrane protein transporters megalin and cubilin. Megalin and cubilin are endocytic receptors that facilitate the uptake of a variety of ligands, including DBP, from the extracellular environment into cells. This process is vital for the reabsorption and conservation of vitamin D metabolites bound to DBP. Megalin and cubilin are predominantly expressed in the renal proximal tubules, but they are also present in other tissues such as the intestines, placenta, and yolk sac, where they mediate the endocytosis of DBP and its associated ligands [4]. The uptake mechanism involves the binding of DBP to megalin and cubilin on the surface of epithelial cells. The DBP-receptor complex is internalized into the cell through clathrin-mediated endocytosis. Once inside the cell, DBP and its bound vitamin D metabolites are trafficked to lysosomes where the vitamin D metabolites are released for further metabolic processing, including conversion to active forms like 1,25(OH)_2_D [5]. Once internalized, DBP associates with the actin cytoskeleton within the cell. This association plays a role in stabilizing and transporting actin filaments, which are critical for maintaining cell structure and function. However, this process can be defective in some diseases of childhood, potentially due to variations in *DBP* genotypes. Certain *DBP* polymorphisms may influence the efficiency of DBP uptake and its interaction with actin, which can impact cellular processes and contribute to disease pathology [4]. For example, in cystic fibrosis (CF), the defective processing of DBP may exacerbate the inflammatory response and tissue damage seen in CF patients. In conditions like acute liver failure, impaired DBP function can result in inadequate scavenging of extracellular actin, leading to vascular occlusion and inflammation [6] (Figure 1).

When exposed to UVB rays from sunshine, the skin produces vitamin D_3_ or cholecalciferol. Vitamin D can also be obtained from diet, such as fortified dairy products, fatty fish, and supplements. Once in the bloodstream, the enzyme 25-hydroxylase transports vitamin D_3_ to the liver where it is converted to 25-hydroxyvitamin D [25(OH)D], known as calcidiol. The enzyme 1α-hydroxylase converts 25(OH)D to 1,25-dihydroxyvitamin D [1,25(OH)_2_D], or calcitriol, which is physiologically active. DBP binds to vitamin D metabolites in the bloodstream, allowing them to travel to other organs. DBP-bound vitamin D metabolites are internalized by cells through interactions with the cell membrane receptors megalin and cubilin, which facilitate endocytosis. Apart from its capabilities of binding and delivering vitamin D, DBP serves various other purposes. It interacts with endotoxins, transports fatty acids, scavenges actin, controls immune responses, and influences the growth and differentiation of cells.

Beyond this essential role of vitamin D transport, the involvement of DBP in a variety of biological processes highlights its importance in health and disease management. DBP has an indirect role in preventing diseases, such as cell injury or death, thus preventing the formation of actin polymers that could lead to vascular occlusion and inflammation. This function is necessary for mitigating the effects of tissue injury and inflammation after trauma and infection. The ability of DBP to modulate macrophage activation, as described in previous studies, contributes to its immune-modulating capabilities, which may alter its ability to combat infections and inflammatory reactions. The significance of DBP in disease control is not limited to the immune system. DBP has been demonstrated to bind fatty acids, suggesting a possible role in lipid metabolism. This activity may affect inflammatory processes mediated by lipid metabolic pathways, as well as how cellular energy is used and stored. Furthermore, DBP may affect cellular growth and differentiation, which are required for appropriate development and repair processes. Another important function of DBP is its interaction with endotoxins. DBP can bind to endotoxins, which are found on the outer membranes of Gram-negative bacteria. This binding reduces the toxicity of endotoxins and prevents their harmful effects on the body. As a result, it supports the body’s natural defenses against bacterial infections. Moreover, DBP interacts with other proteins and hormones, which may have an impact on physiological processes beyond vitamin D and lipid metabolism [5].

## 3. DBP Polymorphisms

DBP is highly polymorphic, with more than 120 described alleles. These variations contribute to significant genetic diversity and influence the protein’s function and its affinity for vitamin D metabolites [5]. There are multiple single-nucleotide polymorphisms (SNPs) in the *DBP* gene on chromosome 4q13.3 that can change the function and amount of DBP. Moreover, rs7041 and rs4588 define the important haplotypes Gc1f, Gc1s, and Gc2 (Table 2). The affinities of the proteins made by the Gc1s, Gc1f, and Gc2 alleles for vitamin D metabolites vary. The SNPs rs7041 and rs4588 are crucial in regulating the amount and bioavailability of vitamin D in the bloodstream. The Gc2 haplotype (rs7041 T and rs4588 A) is linked to lower levels of 25(OH)D than the Gc1s and Gc1f haplotypes. These differences in serum vitamin D levels have the potential to influence immunological response, disease susceptibility, and bone health [5,7]. In addition to these, several other key SNPs in the *DBP* gene have been identified that play substantial roles in serum DBP levels and vitamin D bioavailability: rs17467825, rs222029, rs222020, rs705117, and rs12144344. These genetic variations can affect bone health, immunological function, obesity, and other metabolic disorders. In addition, rs17467825 is linked to differences in fat mass and obesity-related features, emphasizing its function in energy balance and adiposity [8]. Similarly, rs222029 and rs222020 have been connected to bone strength, highlighting their importance in skeletal health [9]. SNP rs705117, discovered in genome-wide association studies, influences serum DBP levels and vitamin D bioavailability, influencing a variety of health consequences [10]. The rare variants p.A246del and p.C311F further illustrate the complexity of the genetic landscape of DBP and its impact on health. Decreased DBP levels caused by the structural deletion p.A246del of the DBP protein impact vitamin D transport and raise the possibility of metabolic issues. The mutation p.C311F modifies the structure of DBP, decreasing vitamin D transport efficiency and raising the risk of autoimmune disorders [11]. The Thr420Lys mutation also reduces the affinity of DBP affinity for metabolites of vitamin D. This lowers the effectiveness of vitamin D transport, which has been connected to a lower level of 25(OH)D [12]. The Asp416Glu polymorphism modifies immune response pathways, which impacts macrophage activation and ultimately leads to chronic inflammation and atherosclerosis [11]. The rs12144344 variation decreases serum DBP levels by altering protein stability and expression, as well as vitamin D transport. This polymorphism has been linked to decreased vitamin D status and an increased risk of osteoporosis [10].

## 4. Impact of Vitamin D and DBP on Childhood Obesity

Vitamin D status has an impact on several aspects of metabolic health, such as fat metabolism. Obese children tend to have low concentrations of vitamin D, which can further exacerbate metabolic dysregulation. A study involving 297 healthy schoolchildren found that 96% of them had low levels of serum 25(OH)D. There was a significant correlation found between vitamin D deficiency and higher waist circumference, insulin resistance (HOMA-IR), and blood pressure. These data indicate a strong relationship between low vitamin D concentrations and indicators of obesity and metabolic syndrome in children [14]. Another study on obese and very obese children showed that 37.5% of the obese and 53.6% of the severely obese were vitamin D deficient. This vitamin D deficiency was associated with greater body mass index (BMI), waist circumference, and lipid levels, demonstrating a direct link between low vitamin D status and metabolic problems [15]. Low vitamin D status was also linked to increased BMI, waist circumference, triglyceride levels, and decreased HDL cholesterol in a study including Korean children. There was a significant increase in the likelihood of obesity, abdominal obesity, and metabolic syndrome in children with the lowest vitamin D status. Children in the lowest quartiles of vitamin D status had significantly higher odds ratios for obesity, abdominal obesity, and metabolic syndrome than children in the highest quartiles [16].

Vitamin D influences adipogenesis, or the process by which pre-adipocytes differentiate into adipocytes. It regulates the expression of genes connected to inflammation and fat formation. A deficiency of vitamin D can impair these activities, increasing fat storage and causing metabolic disturbance. Since vitamin D is fat-soluble, its retention in adipose tissue can also lower its bioavailability, which prolongs vitamin D shortage in obese people [17]. As obesity at any age is widely associated with low vitamin D status, it should be mentioned that one possibility relating low vitamin D status to obesity is simply the ability of the excess adipose tissue to trap vitamin D passing through the circulation, thus limiting the supply for 25(OH)D production in the liver.

A growing body of research indicates that vitamin D regulates oxidative stress and inflammation in fat tissues. These processes play an important role in the formation and advancement of obesity-related disorders. Supplementing with vitamin D reduces the expression of inflammatory cytokines, such as tumor necrosis factor-alpha (TNF-α), interleukin-1 β (IL-1β), and monocyte chemoattractant protein-1 (MCP-1), in the adipose tissue of obese rats on a high-fat diet. This suggests that vitamin D may reduce inflammation in adipose tissue while improving overall metabolic health [18]. In high glucose-treated adipocytes, 1,25(OH)_2_D supplementation increased nuclear factor erythroid-2-related factor 2 (Nrf2) and thioredoxin (Trx) expression while decreasing NADPH oxidase 4 (Nox4) expression, reactive oxygen species (ROS) production, and nuclear factor kappa-light-chain-enhancer of activated B-cells (NF-κB) activation [19]. Vitamin D alleviates oxidative stress in the visceral adipose tissue and mesenteric vessels of obese patients with subclinical inflammation. In vitro treatment with vitamin D reduced oxidative stress markers and improved vascular function, indicating that maintaining an adequate vitamin D status could mitigate obesity-related oxidative stress [20]. Studies conducted on obese mice generated by food have demonstrated that vitamin D supplementation lowers the messenger RNA levels of pro-inflammatory cytokines (e.g., IL-6, MCP-1, and TNF-α) and NLR family pyrin domain containing 3 (NLRP3) inflammasome indicators. This was linked to decreased NF-κB phosphorylation and increased AMP-activated protein kinase (AMPK) activity, indicating that vitamin D may lessen inflammation via the AMPK/NF-κB signaling pathway [21].

Elevated DBP plasma concentrations in overweight or obese women have been associated with a greater fat mass index and lower HDL cholesterol. This shows that high DBP levels may limit lipid metabolism by lowering vitamin D bioavailability, resulting in metabolic abnormalities in obesity [22]. Variations in the *DBP* gene affect vitamin D metabolism by changing the concentration and binding affinity of DBP. Certain *DBP* polymorphisms have been linked to obesity-related traits, such as higher BMI and body fat percentage. For example, the SNP rs17467825 has exhibited high connections with BMI and the percentage of fat mass, particularly in females, showing a genetic predisposition to influence fat metabolism and vitamin D status [8]. *DBP* polymorphisms that decrease the absorption of vitamin D can cause poor fat metabolism and an increased risk of obesity [22]. *DBP* polymorphisms with reduced levels of bioavailable vitamin D can produce insulin resistance and a pro-inflammatory state. Both are critical for the development of metabolic syndrome and obesity [21]. Customized dietary and supplement programs can be made easier with knowledge of how *DBP* polymorphisms affect the risk of obesity. Children with specific *DBP* genotypes may benefit from targeted vitamin D therapy to guarantee proper bioavailability and reduce the risk of obesity. It is expected that children with the Gc2 allele, the TT genotype of rs7041, and the AA genotype of rs4588 will need greater dosages of vitamin D to maintain appropriate serum levels [14].

## 5. DBP and Chronic Disease Risk in Children

### 5.1. Bone Health

Different DBP genotypes (Gc1f, Gc1s, and Gc2) can alter the binding affinity of DBP to vitamin D metabolites, affecting their bioavailability and bone mineral density (BMD) [23]. In one study, the effect of the SNPs rs7041 and rs4588 on BMD and serum DBP concentration in a Mexican population involving 1853 participants from the Health Workers Cohort Study was investigated. Patients with the G allele of rs7041 had higher DBP and BMD compared to the TT genotype. Similar relationships between low BMD, DBP, and the A allele of rs4588 indicate that these polymorphisms have a significant impact on bone health (Table 3) [24].

Certain *DBP* polymorphisms (rs7041 and rs4588 SNPs) are also associated with variations in BMD and the risk of osteoporosis in children [31]. Children with the TT genotype of rs7041 have significantly lower serum 25(OH)D levels compared to those with the GG genotype. This SNP is associated with a reduction in DBP levels, which in turn lowers the bioavailability of vitamin D [13,30]. The G allele of rs7041 has been associated with higher BMD compared to the TT genotype. This suggests that the G allele may confer a protective effect on bone health by maintaining higher levels of bioavailable vitamin D and subsequently better calcium metabolism [24]. Similarly, children with the AA genotype of rs4588 have lower serum 25(OH)D levels compared to those with the CC genotype. This allele has been linked to lower levels of DBP, affecting the overall vitamin D status [32]. The A allele of rs4588 is associated with lower BMD compared to the CC genotype, indicating that this allele might be a risk factor for reduced bone density and potentially higher risk of osteoporosis in children due to lower availability of vitamin D [24,32]. In addition, a study of 231 Finnish children and adolescents discovered that children with the Gc2/2 genotype had the lowest levels of parathyroid hormone (PTH) and serum 25(OH)D. These children also showed lower bone mineral content (BMC) and BMD in the lumbar spine, indicating that this genotype may render them more prone to osteoporosis and decreased BMD in the future [33].

Understanding the familial impact of DBP polymorphisms on bone health can aid in the early diagnosis of children who are predisposed to osteoporosis. This may lead to targeted actions to improve bone health outcomes, such as personalized vitamin D supplementation and lifestyle adjustments. Routine vitamin D and BMD testing in children with high-risk genotypes can aid in the prevention of long-term skeletal issues. Individualized vitamin D supplementation plans may be effective for children with *DBP* genotypes associated with low vitamin D status and BMD. Getting adequate vitamin D helps increase bone mineralization and calcium absorption, reducing the risk of osteoporosis. Promoting exercise and a well-balanced diet rich in calcium and vitamin D can have a significant impact on the bone health of children. Weight-bearing exercises, such as running and leaping, boost BMD and stimulate bone development, which is especially significant for children genetically prone to low bone mineral density.

### 5.2. Autoimmune Diseases

Autoimmune disorders can be influenced by various hereditary and environmental factors. Vitamin D and its binding protein (DBP) have gained attention because of their potential roles in immune regulation. Recent studies have examined the connection between *DBP* polymorphisms and several autoimmune diseases, such as Graves’ disease, type 1 diabetes mellitus, systemic lupus erythematosus (SLE), and juvenile dermatomyositis (JDM) (Table 2).

In a Polish population study, a significant association between Graves’ disease and the Lys allele at codon 420 of DBP was discovered. The Lys allele frequency was higher in Graves’ disease patients than in controls, indicating that Graves’ disease susceptibility is increased by this polymorphism. Furthermore, there was a correlation found between the Lys allele and lower serum levels of 25(OH)D, suggesting a potential association between DBP polymorphisms and vitamin D status in cases of autoimmune disease [12].

DBP levels are lower in patients with type 1 diabetes mellitus who have poor metabolic control. This suggests that DBP may be a biomarker for the severity and progression of the illness. DBP maintains the activity of pancreatic α-cells and regulates the release of glucagon. While DBP is primarily known for transporting vitamin D metabolites, its importance in pancreatic α-cells and glucagon regulation is still not well understood. DBP is highly expressed in the liver and pancreatic α-cells, where it is critical for maintaining α-cell morphology, function, and glucagon secretion. α-cells exhibit significant alterations upon deletion of DBP, such as decreased cell size and hyperplasia. This deletion also affects the conductance of the Na^+^ channel and the ability of low glucose to activate the α-cell, which in turn lowers glucagon release in vitro and in vivo. Mechanistically, the lack of DBP results in reversible changes in the location of glucagon granules, as well as changes in the density and quantity of islet microfilaments. Defects in the activities of β and δ cells were also found, suggesting a more widespread effect on pancreatic cell function. Human investigations have shown that DBP contributes to diabetes mellitus. When the human pancreas was immunostained, it was observed that people with late-onset and chronic type 1 diabetes mellitus exhibited a significant drop in DBP expression, but not those with early-onset diabetes. This suggests that DBP has a function in the formation and maintenance of α-cell activity in diabetes. DBP has a key role in controlling α-cell morphology and function, ensuring adequate glucagon secretion and glucose homeostasis [34].

Type 1 diabetes mellitus may also be facilitated by *DBP* polymorphisms, although there are inconsistent findings. Certain *DBP* alleles have been associated with weakened immune response and elevated risk of type 1 diabetes [35]. This is crucial in the setting of type 1 diabetes mellitus, as the disease progresses due in large part to immunological modulation [36]. Asp/Glu and Glu/Glu genotype frequencies were considerably higher in diabetic participants with detectable IA-2 antibodies [37]. This finding suggests a function for the vitamin D system in the autoimmune process of type 1 diabetes mellitus. In addition, there was a substantial difference in the allele frequency of the DBP HaeIII site between type 1 diabetes mellitus patients and healthy controls. However, there was no discernible transmission disequilibrium for *DBP* alleles in a German group, indicating the need for more research in other populations [38]. A meta-analysis examined the association between two DBP gene polymorphisms (rs7041 and rs4588) and type 1 diabetes mellitus. The results showed no significant association between these polymorphisms and the risk of type 1 diabetes mellitus in overall populations or specific ethnic groups [25]. In White Americans of European origin, no significant association was found between *DBP* gene polymorphisms and type 1 diabetes mellitus [39], despite previous reports linking *DBP* variants with diabetes and prediabetic traits in other populations [37,40]. Also, in Egyptians, there was no discernible correlation between *DBP* polymorphisms and type 1 diabetes mellitus [41].

DBP has also been studied in relation to systemic lupus erythematosus (SLE) and juvenile dermatomyositis (JDM) in children. Low serum 25(OH)D levels were found to be associated with proteinuria and urinary DBP, suggesting that DBP could be a marker for disease activity in children with JDM and SLE [42].

### 5.3. Cardiovascular Disease

A recent study highlighted the effects of DBP and its genetic variations on the development and risk of cardiovascular disease, particularly in the pediatric population. The development of cardiovascular disease is significantly influenced by changes in inflammatory and immunological responses, which are associated with genetic variations in DBP polymorphisms. Certain *DBP* polymorphisms that reduce vitamin D bioavailability can affect endothelial function, arterial stiffness, and inflammatory markers, all of which can have an influence on cardiovascular health [43]. Nitric oxide (NO), a powerful vasodilator produced by endothelial cells, is boosted by vitamin D, which has been shown to have a vasoprotective effect on endothelial function. Vitamin D influences the activity of endothelial NO synthase, which affects NO production. Under pathogenic conditions, oxidative stress decreases NO bioavailability by increasing reactive oxygen species (ROS) generation. Vitamin D counteracts this by suppressing NADPH oxidase, which generates ROS, and increasing the activity of antioxidative enzymes such as superoxide dismutases. Vitamin D inhibits proinflammatory cytokines such as TNF-α and IL-6, protecting endothelial function and suppressing NF-κB signaling [44]. Vitamin D status influences arterial stiffness, which is an independent predictor of cardiovascular disease. Children with low serum 25(OH)D have increased vascular stiffness. This association is critical because arterial stiffness is associated with increased cardiovascular morbidity and mortality. The mechanisms include the role of vitamin D in lowering arterial wall stiffness and increasing vascular endothelial function [45]. Vitamin D deficiency is linked to higher levels of inflammatory markers, which contribute to the development of cardiovascular disease. Increased levels of IL-6 and TNF-α are linked to arterial stiffness and cardiovascular disease and can result from a vitamin D deficit. As these inflammatory cytokines cause endothelial dysfunction and atherosclerosis, it is important to keep vitamin D status sufficient to minimize inflammatory reactions and cardiovascular risk [46].

Different *DBP* polymorphisms vary in their ability to activate macrophages, affecting the ability of the human body to control inflammation and prevent atherosclerosis [47]. *DBP* activates macrophages, leading to an increased release of pro-inflammatory cytokines such as IL-1β, TNF-α, and IL-6. This inflammatory response promotes the progression of atherosclerosis by causing the accumulation of lipids within macrophages and the formation of foam cells, characteristic of atherosclerotic plaques [48]. Variants in the *DBP* gene can affect how important inflammatory pathways are activated in macrophages. For instance, the NF-κB pathway and the cyclic GMP-AMP synthase (cGAS)-stimulator of interferon genes (STING) signaling network, which is essential for inflammatory responses and macrophage lipid uptake, are activated by the TAR DNA-binding protein 43 (TDP-43), which worsens the course of atherosclerosis by encouraging macrophage-mediated inflammation [49]. Macrophage polarization may potentially be impacted by *DBP* polymorphisms. Macrophages can be classified as either M1 or M2 or pro- or anti-inflammatory. A shift towards the M1 phenotype, which is defined by the generation of pro-inflammatory cytokines that support chronic inflammation and atherosclerosis, is linked to specific *DBP* polymorphisms. Asp416Glu and Thr420Lys polymorphisms in the *DBP* gene can influence macrophage behavior and cytokine production, which in turn supports the development of chronic inflammation and atherosclerosis [50]. Extracellular RNA can skew macrophages towards the M1 phenotype, increasing the expression of inflammatory markers such as TNF-α and IL-6, while down-regulating anti-inflammatory genes. This polarization is influenced by *DBP* polymorphisms, suggesting their role in promoting a pro-inflammatory environment [51]. On the other hand, encouraging a change in phenotype towards the M2 type can assist in reducing inflammation and stabilizing atherosclerotic plaques [52]. Epigenetic changes have a significant impact on macrophage activation and inflammation. *DBP* polymorphisms can modify the epigenetic conditions of macrophages, which in turn affects the expression of genes associated with inflammatory reactions. MicroRNAs (miRNAs), specifically miR-223-3p, play a crucial role in regulating the inflammatory response in macrophages and influencing the development of atherosclerosis [53].

Children with lower amounts of bioavailable vitamin D may be more prone to hypertension and other cardiovascular disorders as a result of specific DBP polymorphism. This link emphasizes the importance of monitoring vitamin D status and considering genetic factors when calculating the risk of cardiovascular disease [6]. Obesity, a major risk factor for cardiovascular disease, has been linked to reduced vitamin D status. In this situation, DBP is important because it regulates vitamin D bioavailability. The interplay of vitamin D status, *DBP* polymorphisms, and obesity shows that obese children with specific *DBP* variants may be at a higher risk of cardiovascular problems [54].

### 5.4. Asthma

Asthma is a chronic inflammatory illness characterized by airway hyperreactivity, mucus production, and recurrent wheezing, shortness of breath, and coughing. Asthma has a complex etiology, which includes genetic, environmental, and immunological components. Recent research has highlighted the possible significance of DBP in regulating asthma pathogenesis, particularly in pediatric patients. The immunomodulatory characteristics of vitamin D are thought to influence the onset and severity of asthma. It regulates immunological responses, which may reduce inflammation and improve lung function. Vitamin D insufficiency has been linked to increased asthma incidence and severity, implying that an adequate vitamin D status may help prevent asthma [26]. A Turkish study of 35 children with acute asthma attacks and 32 children with controlled asthma revealed that the acute asthma group had a considerably lower vitamin D status than the controlled asthma group. Low vitamin D status was associated with a considerably higher incidence of acute asthma episodes [55]. In another study including 50 atopic children with mild-to-moderate persistent asthma and 40 healthy children, all aged 6–16 years, asthmatic children have lower levels of total and free 25(OH)D compared to healthy children. DBP levels were comparable between the two groups and no correlation between vitamin D status and asthma control or lung function was observed [56].

Genetic differences in the *DBP* gene may influence the chances of acquiring asthma. Some DBP polymorphisms have been linked to increased production of pro-inflammatory cytokines and activation of macrophages, which can exacerbate the hyperreactivity and inflammation of the airways associated with asthma [57]. In a meta-analysis of 14 studies involving 3278 asthma cases and 3999 controls, a significant link between the rs7041 polymorphism and an elevated risk of asthma in children was found. The presence of the G allele was linked to a higher risk than the T allele presence (OR = 1.18, 95% CI: 1.04–1.33). There was also a weak association between the rs4588 polymorphism and childhood asthma, with the A allele carrying a greater risk than the C allele (OR = 1.12, 95% CI: 1.01–1.24). The polymorphisms rs7041 and rs4588 have an impact on the binding and transport efficiency of DBP, which in turn affects the bioavailability of vitamin D. This, in turn, may have an influence on inflammation and immunological responses, potentially playing a role in the development of asthma [26].

DBP indirectly affects various physiological processes, including inflammation and immunological responses, which are crucial in the context of asthma, by controlling the availability of vitamin D. Another role of DBP is actin scavenging, which is critical during tissue injury and inflammation. Actin polymerization and the resulting tissue damage are prevented by DBP, which binds to free actin released following cell damage. This role is especially crucial in asthma, as destruction and healing of the airway epithelium are ongoing processes. The ability of DBP to reduce inflammation by scavenging actin may help to alleviate some of the hyperresponsiveness and airway remodeling associated with asthma [5]. More studies are needed to investigate the exact mechanisms by which DBP affects the pathophysiology of asthma. Additionally, it is crucial to evaluate the potential impact of DBP on asthma severity and treatment outcomes. Through conducting significant research on the influence of DBP polymorphisms on the incidence and severity of asthma, we can determine who is at risk and implement preventive measures [15].

### 5.5. Allergies

Low serum vitamin D concentrations have been associated with food allergies in children, particularly those with certain *DBP* gene mutations. A case–control study found that genetic variations in the *DBP* gene, particularly rs7041, rs4588, and rs2282679, and vitamin D deficiency may increase the risk of the child of food allergies [58]. Decreased levels of DBP may enhance the bioavailability of vitamin D and regulate immunological responses. Another study found that DBP polymorphisms could alter the association between serum vitamin D status and susceptibility to food allergies. Infants with the GG genotype at rs7041 and low serum 25(OH)D_3_ levels were found to have a higher risk of food allergies compared to those with GT or TT genotypes. Specifically, low serum 25(OH)D_3_ level (≤50 nM/L) at age 1 year was associated with food allergy primarily in infants with the GG genotype [29]. The AA genotype at rs4588 was associated with lower 25(OH)D levels, which may contribute to an increased risk of food allergies. Infants with this genotype and low vitamin D status had higher susceptibility to food allergies. The combination of genotypes from rs7041 and rs4588 could significantly alter the response to vitamin D supplementation and the risk of vitamin D deficiency. Individuals with the TT+CA genotype combination showed the lowest response to vitamin D supplementation, which might predispose them to vitamin D deficiency and associated food allergies [59]. Additionally, the effects of vitamin D and DBP on atopic dermatitis and allergic rhinitis have been examined. Evidence suggests that vitamin D deficiency, which is influenced by DBP, may contribute to the severity and prevalence of several disorders. Children with allergic rhinitis frequently have reduced vitamin D status, which may be impacted by DBP [60]. Individuals with the GG genotype at rs7041 and the AA genotype at rs4588 are more likely to have a lower vitamin D status, which can exacerbate atopic dermatitis symptoms [27]. Children with allergic rhinitis often had a lower vitamin D status, which was significantly influenced by the rs4588 polymorphism [61].

### 5.6. Cystic Fibrosis

DBP has an impact on vitamin D metabolism and the inflammatory process in CF patients. In a study of 40 CF patients and 40 healthy controls, CF patients had significantly lower DBP levels compared to healthy controls. CF patients exhibited reduced levels of 25(OH)D and 1,25(OH)_2_D, consistent with their lower DBP levels. DBP levels had a positive correlation with prealbumin and albumin, suggesting that it may be used as a nutritional measure. DBP levels were correlated with the severity of malnutrition in CF patients. This correlation was demonstrated by the lower serum protein levels and BMI observed in these patients. Individuals with CF who had low DBP levels were more likely to have vitamin D insufficiency and malnutrition. Therefore, DBP has the potential to be a valuable diagnostic tool in the field of nutrition. It could be regularly included as an assessment for CF patients to better address their complex nutritional needs. Further research should focus on validating DBP as a nutritional indicator and exploring its potential in other conditions characterized by impaired nutrient absorption [28].

### 5.7. Acute Liver Failure

When a child develops acute liver failure, the liver cells are rapidly and severely destroyed, allowing intracellular components such as actin to seep into the bloodstream. Elevated levels of free actin can create microthrombi, exacerbating liver damage and leading to vascular issues. DBP helps to alleviate these difficulties by scavenging actin molecules. Newborns who suffer from acute liver failure often have substantially reduced metabolism of vitamin D. Low levels of circulating 25(OH)D are caused by liver failure, which halts the primary process of converting vitamin D to its active form in the liver. Throughout liver failure, DBP transports and regulates vitamin D metabolites to maintain calcium homeostasis and immune system performance. Low DBP levels have been observed in children with acute liver failure, indicating the severity of the disease and a poor prognosis. Monitoring DBP levels may be a useful biomarker for early diagnosis and progression of acute liver failure in pediatric patients. DBP, with its multifunctional role, is a possible therapeutic target in acute liver failure. Therapeutics aiming at increasing DBP levels or function may enhance outcomes by lowering actin-mediated vascular problems and promoting vitamin D metabolism. During acute liver failure, disturbances in vitamin D metabolism are observed. Vitamin D supplementation may be necessary to maintain sufficient levels of bioavailable vitamin D. The supplementation plan should consider binding capacity and DBP levels to guarantee that vitamin D function is successfully restored [6,62].

### 5.8. Celiac Disease and Inflammatory Bowel Disease

Vitamin D deficiency is common in pediatric gastrointestinal diseases such as celiac disease and inflammatory bowel disease (IBD). DBP is involved in maintaining vitamin D status, and its polymorphisms can affect disease severity and treatment outcomes [63]. Specifically, DBP levels correlate with disease activity and inflammation in IBD [64]. The rs7041 GG genotype was associated with a lower vitamin D status and higher disease activity in IBD patients. The TT genotype was found to be protective, and associated with a higher vitamin D status and lower inflammation [65]. Individuals with the AA genotype had lower DBP and 25(OH)D levels, which correlates with more severe disease activity in IBD. This polymorphism is particularly significant in pediatric patients with inflammatory conditions [59]. Patients with the rs7041 GG + rs4588 AA genotype combination had the lowest vitamin D status and the highest disease severity in IBD patients [29]. The rs2282679 polymorphism in the *DBP* gene was associated with lower serum DBP and vitamin D status. This polymorphism has been linked to increased disease severity in pediatric IBD patients, highlighting its role in the inflammatory response and vitamin D metabolism [66].

### 5.9. Kidney Disease

Chronic kidney disease (CKD) is a worldwide health issue characterized by the gradual deterioration of kidney function. The main causes of CKD in children include congenital anomalies of the kidney and urinary tract (CAKUT), glomerular diseases, hereditary and genetic disorders, systemic diseases, and tubulointerstitial diseases. The significance of DBP in kidney diseases is highlighted by its several functions in immunological regulation and vitamin D metabolism [36]. Children with CKD may have low levels of vitamin D due to limited sun exposure, reduced dietary intake, loss during dialysis, and inadequate renal conversion to the active form. Deficiency rates of vitamin D in children with CKD range from 50% to 92%, and they are more prevalent in the later stages of the disease. Individuals with CKD who do not get enough vitamin D may be at a higher risk for developing cardiovascular disease, experiencing poor bone health, and even death. In addition, vitamin D deficiency is associated with growth problems, fractures, bone discomfort, and secondary hyperparathyroidism. For these reasons, it is essential to keep the vitamin D status in CKD patients at an appropriate level to manage these problems and advance general health. While 400 IU of vitamin D is the recommended daily dosage for healthy persons, larger levels may be needed for those with limited sun exposure. Children with CKD should take vitamin D supplements based on their 25(OH)D levels, which should be checked frequently. For CKD stages 3–5, it is advised to measure 25(OH)D levels yearly and to supplement if levels are less than 30 ng/mL [67].

IgA nephropathy, or IgAN, is a common kidney disease characterized by an accumulation of IgA antibodies in the glomeruli. DBP plays a role in the genesis and progression of IgAN. A possible biomarker for anticipating the reaction to irbesartan, an angiotensin receptor blocker used to treat IgAN, has been found in urine DBP levels. DBP is a helpful tool for individualized treatment strategies for IgAN patients, as higher levels of urine DBP have been linked to non-responsiveness to irbesartan medication [68]. Due to its immunomodulatory qualities, DBP helps IgAN regulate its immunological responses. It may have an impact on renal inflammation processes by increasing the chemotactic activity of C5a, a strong inflammatory mediator [69]. The way that DBP interacts with mesangial cells has the potential to impact the inflammatory response. These cells are activated by IgA deposits in the mesangium, which results in the release of pro-inflammatory cytokines including IL-6, which worsen kidney injury and inflammation [70].

DBP plays a significant role in the pathogenesis and progression of lupus nephritis by serving as a biomarker for disease activity and flare prediction, protecting podocytes from autoantibody-induced injury, and influencing renal pathology through its interaction with the vitamin D receptor. DBP is a new urine biomarker for lupus nephritis (LN). According to one study, urine DBP levels are much greater in SLE patients with active LN, and they are strongly correlated with the degree of proteinuria and renal disease activity. Urinary VDBP is a useful measure for prognosis and monitoring in SLE patients because it can predict the onset of proteinuric flare [71]. DBP has also been linked to preventing autoantibody-induced damage to podocytes in lupus nephritis. A study showed that vitamin D mitigates podocyte damage by reducing aberrant autophagy in podocytes through DBP. This illustrates that DBP, like vitamin D, protects podocyte health in LN [72]. The expression of the vitamin D receptor (VDR) in renal tissues of LN patients correlates with disease activity. Reduced expression of VDR, which is controlled by DBP, is associated with worsening kidney damage and disease activity. This emphasizes the importance of vitamin D metabolism in the pathophysiology of LN and the function of DBP in this process [73].

## 6. Monitoring and Managing Vitamin D Status in Children

Obese children often have a vitamin D deficiency, which has been associated with negative metabolic effects like insulin resistance and increased cardiovascular risk. To address these risks, it is important to implement effective intervention strategies for monitoring and managing vitamin D status. It is critical to regularly check the vitamin D status of obese children, which are more likely to be vitamin D deficient, particularly in the fall and winter when there is less sun exposure. Frequent monitoring facilitates prompt intervention and early detection [74]. In a study of North Texas children, obese subjects had much higher vitamin D insufficiency than their non-obese counterparts [75]. Racial and ethnic backgrounds are among the demographic factors that affect vitamin D status. For instance, children of African American and Hispanic descent are more likely to be vitamin D deficient due to higher melanin levels in their skin, which reduces the effectiveness of vitamin D synthesis [76].

Vitamin D supplementation should be customized to the degree of the insufficiency and the needs of the individual. Obese children may require higher doses due to vitamin D sequestration in adipose tissue. A study found that 1000 or 2000 IU of vitamin D_3_ was helpful in increasing serum 25(OH)D levels and associated metabolic indicators in obese children [77]. Supplementation has been demonstrated to improve insulin sensitivity and lipid profiles in obese children. Vitamin D supplementation was linked to lower total cholesterol, LDL-cholesterol, and ALT levels, as well as higher HDL-cholesterol levels [78]. Foods high in vitamin D include egg yolks, dairy products with added vitamin D, and fatty fish. In addition to preventing obesity, encouraging regular exercise can increase sun exposure, which increases the natural production of vitamin D. Physical activity-based therapies were found to lower fat mass and increase vitamin D status in adolescents [79]. Large-scale vitamin D deficiency can be effectively addressed by implementing community and school-based programs that promote outdoor activities and raise awareness about vitamin D. It is critical to educate parents and other caregivers on the importance of vitamin D and how to ensure optimal levels through meals, supplements, and moderate sun exposure.

## 7. Future Perspectives

DBP has a diverse and crucial role in childhood health by regulating bone health, immunological function, and metabolic processes. Future studies should strive to advance our understanding of these roles and investigate potential therapeutic uses. Given the genetic variations in the *DBP* gene that influence vitamin D metabolism, future research should focus on establishing tailored supplementation regimens, which could improve bone health and immunological function in children, especially those at risk of deficiency-related illnesses. More research is needed to better understand the molecular pathways by which DBP regulates many physiological functions. Understanding how DBP interacts with other proteins and pathways may reveal new treatment targets for autoimmune disorders, asthma, and obesity. Investigating the potential of DBP as a biomarker for a variety of pediatric disorders could help improve early detection and intervention efforts. Specific polymorphisms in the *DBP* gene can predict type 1 diabetes in children as well as cardiovascular disease risk. Comprehensive clinical trials evaluating the efficacy of vitamin D supplementation in children with various *DBP* genotypes would give valuable information about the optimal dosage and potential health effects. Furthermore, exploring the possibilities of DBP-targeted medications may reveal fresh approaches to treating juvenile illnesses. For example, adjusting DBP levels or function could be utilized to boost BMD in babies from particular genetic backgrounds or improve immunological responses. In conclusion, a better knowledge of the function of DBP in child health holds great promise for improving pediatric care. Combining targeted medications with personalized approaches based on genetic profiles shows great potential in preventing and managing childhood health issues.

## Figures and Tables

**Table 1:** Functions of vitamin D-binding protein (DBP).

Functions	Description	Ref.
Transport of vitamin D	Binds and transports vitamin D metabolites including 25(OH)D and 1,25(OH)_2_D.	[5]
Immune modulation	Modulates immune responses, influences macrophage activation, and scavenges actin during tissue injury.	[5]
Lipid metabolism	Binds fatty acids, influencing lipid metabolism and energy utilization.	[5]
Bone health	Regulates vitamin D bioavailability, essential for calcium absorption and bone mineralization.	[5]
Inflammation control	Reduces tissue damage during inflammation by binding and scavenging actin.	[5]
Interaction with endotoxins	Binds to endotoxins found on the outer membranes of Gram-negative bacteria, reducing their toxicity.	[5]
Cellular growth and differentiation	Impacts cellular growth and differentiation, necessary for development and repair processes.	[5]
Interaction with other proteins and hormones	Interacts with other proteins and hormones, potentially influencing various physiological processes beyond vitamin D and lipid metabolism.	[5]

Abbreviations: 25(OH)D, 25-hydroxyvitamin D; 1,25(OH)_2_D, 1,25-dihydroxyvitamin D.

**Table 2:** DBP polymorphisms and their health implications.

Polymorphism	Associated Alleles	Impact on DBP Function	Health Outcomes	Ref.
rs7041	Gc1f, Gc1s	Alters DBP binding affinity for vitamin D metabolites by reducing its efficiency	Lower 25(OH)D levels, increased risk of osteoporosis and obesity, higher disease activity in IBD	[5,7,13]
rs4588	Gc1f, Gc1s	Reduces DBP concentration and vitamin D bioavailability, leading to lower circulating vitamin D	Lower bone mineral density, increased susceptibility to type 1 diabetes	[5,7]
rs17467825	-	Influences DBP levels and fat mass by altering DBP gene expression	Higher BMI and fat mass index, metabolic syndrome	[8]
rs222029, rs222020	-	Affects bone strength by modifying DBP levels and its interaction with vitamin D	Altered bone strength, risk of fractures	[9]
rs705117	-	Changes DBP levels and vitamin D bioavailability by influencing gene regulation	Increased risk of cardiovascular diseases	[10]
rs2282679	-	Lowers DBP levels and vitamin D bioavailability by affecting protein binding sites	Higher risk of inflammatory bowel disease	[13]
rs12144344	-	Decreases serum DBP levels	Associated with higher risk of osteoporosis and lower vitamin D status	[10]
Thr420Lys	-	Reduces DBP binding affinity for vitamin D metabolites	Increased risk of Graves’ disease and lower 25(OH)D levels	[12]
Asp416Glu	-	Enhances macrophage activation	Supports chronic inflammation and atherosclerosis	[11]
p.A246del	-	Lowers DBP levels	Increased risk of metabolic disorders	[11]
p.C311F	-	Lowers DBP levels	Increased risk of autoimmune diseases	[11]

Abbreviations: 25(OH)D, 25-hydroxyvitamin D; BMI, body mass index; DBP, vitamin D-binding protein; IBD, inflammatory bowel disease.

**Table 3:** Role of DBP and its polymorphisms in chronic diseases.

Chronic Disease	DBP Role	Key Polymorphisms	Impact of Polymorphism	Ref.
Bone health	DBP regulates vitamin D bioavailability, affecting calcium absorption and BMD.	rs7041, rs4588, Gc2	GG genotype (rs7041) associated with higher BMD. AA genotype (rs4588) linked to lower serum 25(OH)D levels and lower BMD. Gc2/2 genotype associated with lowest BMD.	[24]
Graves’ disease	DBP polymorphisms influence susceptibility and vitamin D status.	Thr420Lys	Lys allele is more frequent in patients and is associated with lower 25(OH)D levels, indicating increased susceptibility to Graves’ disease.	[12]
Type 1 diabetes mellitus	DBP maintains pancreatic α-cell function and glucagon secretion.	rs7041, rs4588	GG genotype (rs7041) and AA genotype (rs4588) are not associated with increased risk of type 1 diabetes mellitus.	[25]
Asthma	DBP regulates vitamin D bioavailability, influences immune responses and inflammation in asthma.	rs7041, rs4588	G allele (rs7041) associated with higher risk of asthma. An allele (rs4588) linked to higher risk of asthma and increased pro-inflammatory cytokine production.	[26]
Allergies	DBP modulates immune responses, impacting susceptibility to food allergies.	rs7041, rs4588, rs2282679	GG genotype (rs7041) and AA genotype (rs4588) associated with higher risk of food allergies. Polymorphisms affect vitamin D status and immune regulation.	[27]
Cystic fibrosis	DBP impacts vitamin D metabolism and inflammatory processes in CF patients.	rs7041, rs4588	Lower DBP levels in CF patients are associated with vitamin D deficiency and malnutrition.	[28]
Inflammatory bowel disease	DBP levels correlate with disease activity and inflammation.	rs7041, rs4588, rs2282679	GG genotype (rs7041) linked to lower vitamin D status and higher disease activity. AA genotype (rs4588) and CC genotype (rs2282679) associated with more severe disease and lower DBP levels.	[29,30]

Abbreviations: 25(OH)D, 25-hydroxyvitamin D; BMD, bone mineral density; CF, cystic fibrosis; DBP, vitamin D-binding protein.
